# Five shared decision-making tools in 5 months: use of rapid reviews to develop decision boxes for seniors living with dementia and their caregivers

**DOI:** 10.1186/s13643-017-0446-2

**Published:** 2017-03-15

**Authors:** Moulikatou Adouni Lawani, Béatriz Valéra, Émilie Fortier-Brochu, France Légaré, Pierre-Hugues Carmichael, Luc Côté, Philippe Voyer, Edeltraut Kröger, Holly Witteman, Charo Rodriguez, Anik M. C. Giguere

**Affiliations:** 1 Department of Family and Emergency Medicine, Laval University, Pavillon Ferdinand-Vandry, Room 2881, 1050 Avenue de la Médecine, Quebec city, QC G1V 0A6 Canada; 2 Quebec Excellence Center on Aging, St-Sacrement Hospital, Room L2, 1050, chemin Sainte-Foy, Quebec city, QC G1S 4L8 Canada; 3 Research Centre on Primary Health Care and Services of Laval University, Quebec City, Canada; 4 CHU de Québec Research Centre, Quebec City, QC Canada; 5 Faculty of Nursing Sciences, Laval University, Quebec City, QC Canada; 6 University Laval Faculty of Pharmacy, Quebec City, Canada; 7 McGill University Department of Family medicine, 5858 Côte-des-Neiges, Suite 300, Room 328, Montreal, QC H3S 1Z1 Canada; 8 St. Francois d’Assise Hospital, 10, rue de l’Espinay, Quebec city, QC, Room 2122-Z, 1050 Avenue de la Médecine, Quebec City, QC G1L 3L5 Canada

**Keywords:** Knowledge translation, Costs, Decision support technology, Patient decision aid, Evidence summary, Policy, Knowledge tools, Scoping review, Rapid review

## Abstract

**Background:**

Decision support tools build upon comprehensive and timely syntheses of literature. Rapid reviews may allow supporting their development by omitting certain components of traditional systematic reviews. We thus aimed to describe a rapid review approach underlying the development of decision support tools, i.e., five decision boxes (DB) for shared decision-making between seniors living with dementia, their caregivers, and healthcare providers.

**Method:**

We included studies based on PICO questions (Participant, Intervention, Comparison, Outcome) describing each of the five specific decision. We gave priority to higher quality evidence (e.g., systematic reviews). For each DB, we first identified secondary sources of literature, namely, clinical summaries, clinical practice guidelines, and systematic reviews. After an initial extraction, we searched for primary studies in academic databases and grey literature to fill gaps in evidence. We extracted study designs, sample sizes, populations, and probabilities of benefits/harms of the health options. A single reviewer conducted the literature search and study selection. The data extracted by one reviewer was verified by a second experienced reviewer. Two reviewers assessed the quality of the evidence. We converted all probabilities into absolute risks for ease of understanding. Two to five experts validated the content of each DB. We conducted descriptive statistical analyses on the review processes and resources required.

**Results:**

The approach allowed screening of a limited number of references (range: 104 to 406/review). For each review, we included 15 to 26 studies, 2 to 10 health options, 11 to 62 health outcomes and we conducted 9 to 47 quality assessments. A team of ten reviewers with varying levels of expertise was supported at specific steps by an information specialist, a biostatistician, and a graphic designer. The time required to complete a rapid review varied from 7 to 31 weeks per review (mean ± SD, 19 ± 10 weeks). Data extraction required the most time (8 ± 6.8 weeks). The average estimated cost of a rapid review was C$11,646 (SD = C$10,914).

**Conclusions:**

This approach enabled the development of clinical tools more rapidly than with a traditional systematic review. Future studies should evaluate the applicability of this approach to other teams/tools.

**Electronic supplementary material:**

The online version of this article (doi:10.1186/s13643-017-0446-2) contains supplementary material, which is available to authorized users.

## Background

Knowledge syntheses may serve to summarize the results of primary studies and may support the development of knowledge tools/products that are later implemented in practice, evaluated, and sustained [1]. Rapid reviews are a type of knowledge synthesis used to produce timely information to support health decisions [2]. Compared to more traditional knowledge synthesis methods, such as systematic reviews according to Cochrane [3], rapid reviews shorten or omit some of the steps from systematic reviews to achieve results in a more timely manner [2, 4, 5]. Depending on the total body of literature in the area, systematic reviews can take from 6 to 71 weeks (average 32 weeks) to complete [6], which is often an unacceptable delay in the context of urgent or pressing decision-making. Rapid reviews are thus being used increasingly to address the needs of policy-makers, administrators, or clinicians [7] when resources or time are lacking to conduct a conventional systematic review [2, 8, 9]. There is currently no standard or universal methodology for rapid reviews [10]. A recent study described six different methodological approaches to conducting rapid reviews [11], with content and format generally adapted to their intended use [5, 12].

Knowledge syntheses are extremely valuable in the development of shared decision-making tools. The shared decision-making process is recommended whenever evidence is insufficient to clearly support one health option over the others, or when proof of benefit is more or less counter-balanced by proof of harm [13]. During a shared decision-making process, the clinician and patient discuss the best evidence on the benefits and harms of each of the options available to address a patient’s health problem [13, 14]. Evidence is then typically presented to patients in the form of a patient decision aid [15] or other shared decision-making tools such as the decision box [16, 17] or the Option Grid [18]. Shared decision-making tools should meet the International Patient Decision Aid Standards (IPDAS) [19, 20], which imposes prerequisites on the systematic review process on which the decision aid will be based. For example, the shared decision-making tool should synthesize the evidence regarding all health options available to a patient facing a specific health problem. Consequently, several syntheses are often required to populate a single tool. In addition, because the shared decision-making tool should present positive and negative features of each option with an equal amount of details, less robust sources of information often have to be used to present the harms of health options that are often underreported in scientific sources. Table 1 sets out the impacts of meeting IPDAS standards for shared decision-making tools on the review processes to synthesize evidence.

**Table 1:** Impact of certification standards for shared decision-making tools on the review process

Selected standards for the certification of shared decision-making tools (IPDAS)	Impact on the review process
The tool describes the options available for the index decision (level 1).	▸ Several interventions need to be reviewed to design a single shared decision-making tool.
The tool describes the positive and negative features of each option in equal detail (levels 1 and 2).	▸ Less robust sources of information need to be used when there is a lack of evidence or detail (e.g., harms of an intervention).
The tool allows users to compare the negative and positive features of available options (level 3).	▸ All probabilities need to be transformed into absolute risks with a common denominator, to allow the comparison of benefits and harms, as well as the various options.
The tool enables the comparison of outcome probabilities using the same denominator (level 3).	

Although rapid reviews have been used in several studies [4, 5, 21, 22], little is known about their use in the development of shared decision-making tools. Consequently, we describe a rapid review approach underlying the development of five decision boxes (DB) designed to meet the decision-making needs of community-dwelling seniors living with dementia, and of their caregivers and healthcare providers. The five decisions covered were prioritized in an earlier Delphi study of experts (Poirier A, Philippe Voyer, France Légaré, Michèle Morin, Holly Witteman, Edeltraut Kröger, Bernard Martineau, Charo Rodrìguez, Giguere AMC: Caring for seniors living with dementia means caring for their caregivers too, in preparation) as some of the most difficult decisions frequently faced by this population (list of decisions: Table 2). In the present study, we specifically describe: (1) the rapid review steps, and (2) the resources and time required for the review process and DB prototype design.

**Table 2:** Inclusion criteria for each decision box (legend: *SwD* seniors living with dementia, *P* participants, *I* intervention, *C* comparative group, *O* outcomes)

Titles of the decision boxes (DBs)	Inclusion/exclusion criteria (PICO question)
DB1. Choosing a support option to decrease the burden of caregivers of SwD or to improve their quality of life	(P) Informal caregivers of SwD, (I) any intervention, (C) usual care or any alternative intervention, (O) burden or quality of life measured with validated scale, or any validated measure related to any of the quality of life domains
DB2.Choosing a treatment to manage agitation, aggression, or psychotic symptoms in SwD	(P) SwD, (I) any intervention, (C) usual care or any alternative intervention, (O) psychotic symptoms, aggression, agitation
DB3. Deciding whether or not to stop driving following a diagnosis of dementia	(P) SwD, (I) stopping driving, (C) continuing to drive, (O) any outcome
DB4. Choosing an option to maintain or improve the quality of life of SwD	(P) SwD, (I) any intervention, (C) usual care or any alternative intervention, (O) quality of life measured using a validated scale or any validated measure related to any of the quality of life domains
DB5. Deciding whether to prepare advanced directives and a protection mandate following a diagnosis of dementia	(P) SwD, (I) preparing advanced care directives or any other legal protective supervision measures, (C) usual care or any alternative intervention, (O) any outcome

## Methods

### Study design

This is a descriptive study of the steps to implement a rapid review approach as part of the creative process of developing five decision boxes (henceforth DB).

### Time resources and expertise

We defined the successive project steps and asked reviewers to self-report the time required to complete each of these steps, at the end of each working day, in a Google Drive worksheet (Google sheets) [23]. The first author (MAL) collected this data for each DB and from all reviewers. We used a Dropbox platform to share project documents.

### Rapid review process

We used four specific strategies to shorten the review process. The first strategy involved having a single reviewer conduct literature searches and study selection. The second strategy consisted of searching a limited number of databases selected for content relevance with regard to each of the studied decisions. The third strategy consisted of using an “overview of reviews” approach, by searching for secondary literature sources first. The fourth strategy consisted of engaging several reviewers in data extraction and having them mutually review their results as part of the process to minimize the risk of one reviewer misinterpreting the results extracted. The following sections provide more details on the strategies used.

#### Step 1: searching for evidence

**Fig. 1:**
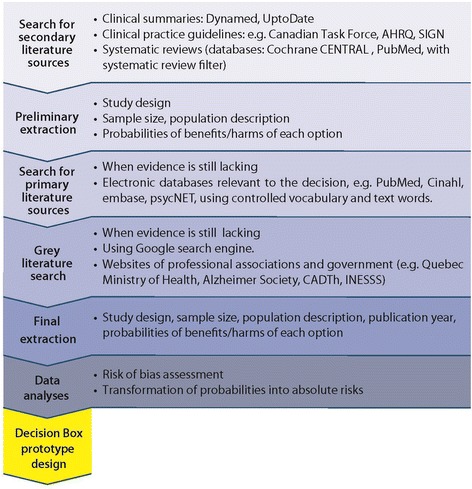
Steps of the rapid review process leading to the design of the decision box prototype

For each DB, a single reviewer conducted a two-stage literature search. The reviewer consulted an information specialist to develop the search strategies (Fig. 1). The reviewer initially searched for secondary sources of literature and, once data was extracted from these sources, searched for primary studies to fill any gaps in the evidence relative to a particular health option. The searches comprised both grey literature sources and academic databases.

##### Search for secondary sources of literature

We searched two online clinical summaries: Dynamed and UpToDate. We chose Dynamed because these summaries are accessible without costs at our university, and UptoDate out of habit, since we used to access them at no cost. We also searched clinical practice guidelines [e.g., Canadian Neurological Sciences Federation, CCCDTD4, Canadian Task Force, AHRQ, Scottish Intercollegiate Guidelines Network (SIGN), National Institute for Health and Care Excellence (NICE), American Academy of Neurology], and systematic reviews. We conducted the search for systematic reviews in the Cochrane Central Register of Controlled Trials (CENTRAL) and in PubMed (NLM) using the built-in systematic review search filter (Additional file 1: Search strategies). These searches allowed us to list most of the available health options to be considered for each of the five targeted decisions. Because the health options may not all be reported in scientific sources of information, and to ensure the comprehensiveness of this list of options, several discussion, and brainstorming sessions were conducted with the principal investigator (PI) responsible for the project (AMCG) first, and then with all the review team members. We then extracted data from these sources and critically reviewed the available evidence for each health option.

##### Search for primary studies

Where evidence was lacking from secondary literature sources, we searched for primary literature sources using the electronic databases relevant to each decision, including PubMed, The Cochrane Library, Cinahl, EMBASE, and APA PsycNET. The search strategies combined keywords from controlled vocabulary as well as free text to optimize the identification of relevant reports. We also scanned the references cited in the included primary and secondary literature sources.

When we found no benefits or harms related to a health option in these academic databases, we conducted additional searches in grey literature using the Google™ search engine. We searched the websites of professional associations and government (i.e., Quebec Ministry of Health, Ministry of Justice, Alzheimer Society, CADTh, Nutt and Sharpe, Public curator—curateur.gouv.qc.ca, INESSS, educaloi.qc.ca).

#### Step 2: identifying eligible studies

The criteria for inclusion of studies in each of the five reviews were set iteratively based on preliminary evidence to clarify the scope of the review, notably the population of interest, the health options, and the outcomes of interest. These elements were gathered in a DB preliminary template (www.decisionbox.ulaval.ca). The template was initially submitted to the PI. Then, a brainstorming session with all the reviewers involved in the design of a DB allowed us to explore whether further clarifications were required before launching a comprehensive literature search.

For each DB, we described inclusion criteria based on a PICO question (Population, Intervention, Comparison and Outcome) (Table 2) and on study design, giving priority to systematic literature reviews and, when no review was available, to randomized controlled trials (RCT). In the absence of robust trials, we included experimental studies other than RCTs, then qualitative studies, and in the last resort, non-scientific publications (e.g., legal texts, government reports, expert opinions). We included studies published in English, French, and Spanish and excluded studies in any other language.

A single reviewer screened the references retrieved using a bibliographic management software (Endnote version X7.7.1, Clarivate Analytics). Screening was conducted based on the title and abstract first, then on the full text. When several sources were relevant, we selected the most relevant by extracting key elements to compare their applicability to our context (descriptions of the population and intervention) and their quality (e.g., study design, number of studies included, and presence of a meta-analysis for the reviews, dates covered, population, outcomes, intervention description) in an Excel spreadsheet (2016 version, Microsoft Corporation).

We did not record the reasons for excluding a reference.

#### Step 3: extracting data

From the included studies, a single reviewer extracted the study design, sample size, characteristics of the study population, year of publication, and probabilities of experiencing benefits or harms for each studied health option in an Excel spreadsheet. If a source reported a body of evidence amenable to meta-analysis but the authors did not conduct any, then we performed the meta-analysis. The PI or the study coordinator (BV) verified the extraction, ensuring it met the team’s internal standards of quality, often coaching the reviewer in knowledge synthesis methodology during this stage.

#### Step 4: assessing the quality of the included studies

Quality of evidence was assessed for each benefit or harm extracted from quantitative studies using the GRADE approach (Grading of Recommendations Assessment, Development and Evaluation) [24–26]. Assessments were based on four criteria (risk of bias, precision, consistency, and directness of study results) and led to a level of confidence in the results rated as “high,” “moderate,” “low,” or “very low.” When an included systematic review reported GRADE assessments, we reported these assessments directly in the DB. When an included systematic review did not report any GRADE assessment but reported risks of bias of the primary studies included, then we used those risks of bias assessments to complete the GRADE assessment. When a systematic review did not report any assessment of the quality of evidence, we completed both risks of bias and GRADE assessments. We assessed the risks of bias of RCTs and before-and-after trials using the Cochrane Risk of Bias Tool [8]. For observational studies (case-control and cohort), we used the Newcastle-Ottawa Scale [27]. For qualitative studies or non-experimental information sources, we did not conduct any quality assessment to speed the review process.

For each benefit and each harm, a first reviewer completed the GRADE and risk of bias assessments, which were verified by a second experienced reviewer (BV). Any discrepancies between reviewers were resolved by consensus first, or in a team meeting with the PI, if a consensus could not be reached. These meetings served as a coaching opportunity to further the expertise of the team in knowledge synthesis methodology. We produced general directives for GRADE assessments as the result of this process and published them for the team in a Google Drive folder.

#### Step 5: analyzing data

Whenever possible, the probabilities extracted were transformed into absolute risks (AR) and absolute risk differences (ARD = AR in intervention group − AR in control group). For dichotomous outcomes, we extracted the AR in the intervention and control groups where available. When we found a statistically significant effect and the raw data was not available to compute AR and ARD (e.g., when only odds ratio, relative risk, or hazard ratio were available in a report), we mentioned that the intervention had statistically significant positive impacts, without providing any figures on the extent of this impact. For continuous outcomes, we extracted the standardized mean differences (SMD) presented in the articles where available. If the SMD was not reported in the article, we calculated it using the means and standard deviations in the intervention and control groups. Using a published methodology [28], we then converted the SMD to an effect size (Cohen’s d), which was in turn used to calculate the correlation coefficient (*r*) [*r* = *d*/√ (*d* ^2^ + 4)]. Then, we used correlation coefficient (*r*) to calculate the success rate of the intervention group (0.5 + *r*/2), the success rate of the control group (0.5 − *r/*2), and the ARD using a web calculator [13]. These transformations were carried out by a single reviewer and were verified by a second reviewer (BV) and a biostatistician (PHC).

To determine the time necessary to complete the knowledge syntheses, we calculated the number of work hours at each step. The length of time required for training and coaching was included in these calculations. We converted these figures into an equivalent number of weeks, using 35 h/week as the reference. We then calculated descriptive statistics for these figures. We also estimated the costs associated with hiring each reviewer, taking into account their salary (range: C$10.08 to 44.50 per hour, including benefits). We estimated the costs of graphical design of the BDs based on an hourly rate of C$60 per hour. Importantly, we do not report the costs associated to the time of the PI, information specialist and biostatistician, and neither do we report the costs of ordering of references, as our research centre and university provide these resources at no costs.

#### Step 6: expert validation

Between one and four experts reviewed each DB content for validation purposes. Epistemic experts were selected, i.e., people with “a capacity to provide strong justifications for a range of propositions in a domain” [29]. Some of the experts selected also had performative expertise, i.e., “a capacity to perform a skill well according to the rules and virtues of a practice” [29]. We summarized all the comments received for each DB in a single document, noting our decision whether to modify the DB to address each comment and returned the summary document to the experts to keep them engaged in the process until they are involved again at the tailoring and updating steps (not reported here).

### Decision box prototype design

The results from the five knowledge syntheses were reported in DB templates, which are designed in such a way as to help weigh the benefits/harms of all options in light of the patient’s individual health status, as previously published [16, 17, 30]. The DB is tailored to the needs of each type of user, as it includes a version designed for clinicians (C-DB) and a lay version for patients/caregivers (P-DB). The C-DB is designed as a continuing education activity. It provides clinicians with scientific information that they can review carefully prior to patient consultations. It is more succinct than the P-DB and allows clinicians to appraise the evidence critically by describing the design and participants of included studies and by synthesizing study limitations using the GRADE approach [31]. The P-DB, on the other hand, is designed for use during consultations to encourage discussion and to be left with the patient and caregiver for review afterwards. It is distinct from the C-DB as it presents the information in complete sentences, in non-technical language, features larger font sizes, and comprises a value clarification exercise and an instrument to screen for decisional conflict. The P-DB design is inspired by the Ottawa Decision Support Framework [32] as well as current international standards for decision aids [21].

Once the content was adapted to fit into each template, we sent it to a graphic designer who prepared printable versions of each decision box.

## Results

### Description of studies

#### Results of the search

Depending on the DB, we identified between 104 and 406 references and selected 15 to 26 of them (Table 3). The number of references screened was highest for DB1 (support for caregivers), and the number of selected studies was highest for DB2 (agitation, aggression, and psychotic symptoms). We identified between 2 and 10 health options per DB and extracted between 11 and 62 health outcomes per DB (Table 3). Notably, we found more benefits than harms in DB5 (advanced directives). By contrast, we found more harms than benefits in DB3 (driving cessation). For the other DBs, the number of outcomes was generally evenly divided between harms and benefits.

**Table 3:** Description of the references screened, studies included, options studied, and outcomes retrieved for each decision box

	DB1-caregiver	DB2-agitation	DB3-driving	DB4-QoL	DB5-advanced directives	TOTAL
Number of references identified	406	237	104	252	111	1010
Number of studies included	23	26	16	20	15	100
Systematic reviews	12	15	2	3	1	33
Non-systematic reviews	1	2	0	1	0	4
RCTs	3	5	0	14	2	24
Non-randomized trials	5	2	14	0	3	24
Qualitative studies	1	0	0	2	0	3
Non-scientific publications	1	2	0	0	9	*12*
Number of health options	7	10	2	5	2	25
Number of health outcomes	62	58	11	29	17	177
Total number of benefits	42	29	3	23	10	107
Total number of harms	20	29	8	6	7	70
Number of GRADE quality assessments	47	45	11	12	9	124
Number of outcomes for which a GRADE evaluation was not possible	15	13	0	17	8	53
Number of experts who provided comments	3	5	3	4	3	18

#### Included studies

The number of included studies and the proportion of studies of each type (reviews, RCTs, non-randomized trials, qualitative studies, and non-scientific publications, e.g., legal texts, government reports, and expert opinions) varied across the five DBs (Table 3).

Of the 33 systematic reviews included in the 5 DBs, 24 (73%) included a meta-analysis. Of the 12 non-scientific publications cited in the DBs, 9 (75%) were included in DB5 (advanced directives). DB4 (quality of life) was mostly based on systematic reviews and RCTs while DB3 (driving cessation) was based on 2 systematic reviews and 14 non-randomized controlled trials. The non-scientific publications comprised expert opinion (*n* = 1), reports (*n* = 2), books (*n* = 1), websites (e.g., website of the *Curateur public du Quebec* responsible for protecting incapacitated individuals) (*n* = 1), and legal documents (*n* = 7). Three qualitative studies were used, two in DB4 and one in DB1.

### Extraction

The number of extracted health outcomes was highest for DB1 (*n* = 62), followed by DB2 (*n* = 58).

### Quality assessment using the GRADE approach

Overall, we completed 124 GRADE quality assessments, with numbers of assessments ranging from 9 to 47, depending on the DB (Table 3). For 53 of the outcomes, we did not complete any GRADE assessment, either because the outcomes were reported in qualitative research studies (17 outcomes) or in non-scientific literature (20 outcomes), or because the outcome was not supported by any evidence (e.g., time required to attend psychotherapy) (16 outcomes).

### Time required

We separated the activities leading to the creation of the prototypes into two: (a) the rapid review process, and (b) decision box prototypes design. We defined the rapid review process as the period starting when we initiated the preliminary search of the literature to the end of the integration of experts’ inputs. We defined the decision box prototype design as a stage that included adapting content (e.g., reading level) and tailoring graphic design to each type of user (clinicians and patient/caregivers). The entire project required a total of 3300 h. Of these, 2800 were required for the rapid review process and 500 for the DB prototypes design.

Depending on the DB, the rapid review process required an equivalent of 7 to 31 weeks for a single reviewer working full-time (Table 4). On average, an equivalent of 19 weeks (SD = 10) full-time was required to complete a single rapid review, representing approximately 4.8 months. These estimates comprise the time required for question refinement, inclusion criteria selection, search strategy development, but do not comprise the time of the PI, biostatistician, and information specialist. The final data extraction step was the most time-consuming, taking an average of 8 weeks for a single DB (SD = 6.8). The rapid review in support of DB5 (advanced directives) required the least time (7 weeks) of the five. The rapid reviews for DB1 (support for caregivers) and DB2 (agitation, aggression, and psychosis) required the most time (30 and 31 weeks, respectively). Depending on the DB, the experts’ comments were incorporated into the DB within 0.8 to 1.6 weeks. Based on these times, and excluding the time spent by the PI and biostatistician, we estimated that the cost of a single review ranged from C$1925 to C$30,130 (mean ± SD; C$11,646 ± 10,914).

**Table 4:** Number of weeks required for each step of (a) the rapid review process, and (b) Decision box prototypes design for each decision box (considering 35 h/week for a single person)

Step	DB1-caregiver	DB2-agitation	DB3-driving	DB4-QoL	DB5-advanced directives	Mean (SD)
A/Rapid review process						
Search for secondary literature sources	0.4	0.4	0.7	0.8	0.6	*0.6* (*0.2* *)*
First selection (secondary sources)	0.4	0.8	0.2	0.6	0.4	*0.5* (*0.2*)
Preliminary extraction	2	0.8	0.9	3.4	0.6	*1.5* (*1.0*)
Search for primary literature sources and grey literature	2.4	2.6	1.0	2.4	0.8	*1.8* (*0.8*)
Second selection (primary studies)	2.0	2.3	1.0	2.0	1.0	*1.7* (*0.5*)
Final extraction	14.8	17.3	1.3	6.0	1.1	*8.1* (*6.8*)
Quality assessment	3.0	3	1.4	1.0	0.8	*1.8* (*1*)
Redaction	1	1	1	1	1	*1* (*0*)
Data analysis and transformation	3	1.2	0.8	0.4	0.4	*1.2* (*0.97*)
Integration of expert input and formatting of references	0.8	1.6	0.8	0.8	0.8	*1* (*0.3*)
Total number of weeks (A, review processes)	***30***	***31***	***9***	***18***	***7***	***19*** (***10***)
B/decision box prototype design						
Adjustments to reading level and content	0.4	0.7	0.1	0.7	0.6	*0.5* (*0.2*)
Graphic design	1.3	1.0	0.5	0.3	0.3	*0.7* (*0.4*)
Total number of weeks (B, prototype design)	***1.7***	***1.7***	***0.6***	***1.0***	***0.9***	***1*** (***0.4***)
Total time (A + B)	***32***	***33***	***10***	***19***	***8***	***20*** (***11***)

During the decision box prototype phase, a senior research associate initially adapted the reading level and content to each type of user, which required 4.5 to 25.5 h per DB (mean ± SD, 18 ± 8 h). A graphic designer then created the printable color version of each DB, requiring on average 25 h/DB (SD: 14 h) (range: 11 to 47 h/DB). In total, we estimate that an average of 20 weeks (SD: 11) was required to complete both the review and the prototype design processes for each DB (range: 8 to 32 weeks, depending on the DB) (Table 4). Based on these times, we estimate that the cost of developing a prototype based on the rapid review results ranged from C$1194 to C$3304 (mean ± SD; C$2188 ± 982).

Overall, the costs of the whole of the activities comprising the rapid review process and decision box prototypes design ranged from C$3581 to C$27,335 (mean ± SD; C$13,429 ± 9024).

### Human resources and expertise

Ten reviewers were responsible for reviewing a specific DB, often in teams of two for a single DB (Table 5). They conducted literature searches, study selection and extraction, and drafted the DB content. To ensure the tools would be relevant to users, three of the reviewers were potential users of the DB, as they were undergraduate medical students. The reviewers were supervised by the PI and the project coordinator (BV) who holds a Doctorate in epidemiology (BV).

**Table 5:** Human resources required

Role	Training and expertise	Responsibilities
Principal investigator, project supervisor and coordinator (2 days/week)	▸ PhD ecotoxicology; professor in the Department of Family Medicine and Emergency Medicine; systematic review author; Cochrane-certified trainer for systematic review authors	▸ Plan the review process ▸ Train team members in systematic reviewing (in groups and individually) ▸ Organize team meetings ▸ Liaise with the graphic designer ▸ Critically revise all DB content
Project coordinator	▸ Research associate (pharmacist, PhD epidemiology) with experience in the conduct of one systematic review	▸ Act as primary resource to all team members ▸ Liaise with the biostatistician ▸Transform probabilities ▸ Revise the GRADE assessments
Reviewers (*n* = 10)	▸ 4 undergraduate students (1 in psychology, 3 in medicine) ▸ 2 PhD students (psychology, clinical and biomedical sciences); each of them with experience in the conduct of one systematic review ▸ 1 postdoctoral fellow (PhD neuropsychology) with experience in the conduct of one systematic review ▸ 3 research assistants (PhD nutrition sciences, MSc public health, PhD epidemiology)	▸ Conduct all review steps ▸ Liaise with information specialist ▸ Work in teams for larger DBs
Graphic designer	▸ Specialization in the design of professional training and health promotion materials	▸ Prepare the final graphic version of each DB
Biostatistician	▸ MSc biostatistics	▸ Confirm data transformation
Experts (*n* = 10)	▸ 5 healthcare providers: 1 physician (geriatrician), 2 nursing experts, 1 pharmacist, 1 social worker ▸ 1 informal caregiver ▸ 2 managers of community-based or institutional organizations	▸ Provide input and make suggestions to improve the DBs

We asked content experts (physician-geriatrician, nurse, informal caregivers, managers of community-based or institutional organizations, pharmacist, and social worker) to review and provide feedback on the content of each DB before beginning work on the graphic design (Table 5). The content of each DB was reviewed by between 3 and 5 experts (Table 3). They made various comments, including suggestions about new references to consider; information to add about the evidence presented, such as drug doses and length of interventions; additional interventions; benefits or adverse effects of an intervention; and the possibility of transforming the probabilities of certain benefits and harms. For DB2 (agitation, aggression, and psychotic symptoms), three experts had serious concerns about the evidence presented relative to medications and asked that the increased risk of death in patients taking atypical antipsychotics be added. One of these experts also asked that we include a section about the conditions that need to be verified before starting any pharmacological treatment for behavioral and psychological symptoms of dementia (BPSD). The experts’ concerns regarding the current inappropriate drug prescription for BPSD in primary care led us to remove the evidence on the impact of these options from the DB. In DB4 (quality of life), two experts suggested including a number of additional treatment/intervention options. Other minor comments by the experts consisted of grammar corrections, rewording of some sentences to improve understanding, and removal of information they considered less relevant (e.g., they suggested removing the use of a therapeutic robot to improve quality of life as it is not yet available in Canada for community-based seniors with dementia).

### Project management

During the course of the study, the reviewers who lacked training in the conduct of systematic reviews attended some formal training of the Cochrane for systematic review author, and team workshops led by the PI on GRADE study quality assessment. These basic training sessions were subsequently complemented with informal coaching by the PI and study coordinator (BV). At the start of each new stage of the review (e.g., selection, extraction), each reviewer’s work was revised iteratively by the PI and the coordinator, in the presence of the reviewer, ensuring it met the team’s internal standards of quality, so as to enhance the team’s expertise and the quality of their work.

### Data synthesis for decision boxes

To facilitate understanding of the probabilities presented by users, we presented numeric estimates of the effect of an intervention into absolute risks whenever possible. A number of the AR were already available in some of the included studies, however, we had to calculate the SMD or absolute risk differences for others. We did not transform 107 outcomes (between 1 and 37 per DB), either because the results were not statistically significant or because they were non-quantifiable. This last aspect is particularly important for DB5, which presents several legal aspects that have not been reported on in scientific studies and could thus not be quantified with regard to risks and benefits. Since some of the probabilities could not be transformed, we reported data in absolute risk reduction for 70 outcomes (25 for DB1, 22 for DB2, 10 for DB3, 8 for DB4, and 5 for DB5) out of the 177 reported.

## Discussion

In this study, we describe an approach for conducting rapid reviews to populate shared decision-making tools. We examine the resources required to complete five specific rapid reviews conducted using this approach for patients affected by dementia. We highlight the requirements of shared decision-making tools, which have repercussions on the review process underpinning these tools, notably the need to review the evidence on all available health options for every tool and the need to transform probabilities according to best practices in risk communication. We also describe several strategies to speed up the review process, notably a preliminary search of secondary literature sources and execution of several review steps by a single reviewer with verification by a second reviewer. For the five specific rapid reviews in our study, we describe how a team of ten reviewers with various backgrounds and expertise, directed by a PI with extensive experience in review methodology and coordinated by an epidemiologist, performed the reviews within 7 to 31 weeks, depending on the review. The study also describes the specialized resources that are called on at each stage: an information specialist for literature searches, a biostatistician for GRADE assessments and data transformations, and a graphic designer to create the printable tools. The study shows wide variations in the number of resources across reviews, likely due to variations in the number of outcomes reviewed and due to the availability of systematic reviews with meta-analyses to describe the benefits and harms of each of these options.

The proposed rapid review approach enabled the development of five tools for shared decision-making—decision boxes—that users (clinicians, patients, caregivers) may find easy to understand. Indeed, we propose a strategy to improve user understanding of the benefits and harms of each of the health options by transforming all probabilities into absolute risks. Absolute risks have been shown to improve statistical literacy and understanding of probabilities in both doctors and patients [33]. Overall, the approach documented herein is an example of a specific use of rapid reviews, the need for which has been expressed by health system decision-makers (Ministry of Health and Social Services in Quebec, Canada) [34, 35]. Our approach also offers the possibility to present quality assessments of the included studies and to focus on topics prioritized by practicing health care providers; two elements deemed crucial in decision-making [35]. Moreover, the involvement of content experts was very valuable to ensure the applicability of the results within the local context of implementation.

Among the strategies that we used to shorten the review process, we chose to search a limited number of databases selected for content relevance. Limiting the number of databases searched has been previously highlighted as a potential cause of bias [36], however, a recent study reported that a vast majority of relevant studies can be found within a limited number of databases and that there are no changes in results when the search comprises more databases [37]. Some researchers have also previously reported that manual searches of reference lists and contacts with experts may be a more effective way to acquire information on all performed trials more quickly than through comprehensive bibliographic database searches [38, 39], since published reviews never include all trial results [40]. In a study on the attitudes and perceptions of users toward rapid reviews, users perceived that a well-conducted rapid review might produce evidence as valid as that produced through a more traditional systematic review approach [41]. To the best of our knowledge, our strategy to use an “overview of reviews” approach, by searching for secondary literature sources first, has not been reported previously in the context of rapid reviewing. Our strategy to engage several reviewers at the time so they can mutually review their results allowed us to train the reviewers while validating review results and normalizing our approach across several tools/reviewers. Some of these strategies have been previously reported [22].

The average time required to complete the 5 reviews was 19 weeks, which is similar to the time reported in rapid reviews [12, 42]. Notably, a recent study reports a median review time of about 15 weeks per review, based on empirical observations made when completing 12 rapid reviews on various topics related to policy, system, and processes of care [42]. We are aware of a single study reporting the time required to develop 12 patient decision aids based on the results of Cochrane systematic reviews, which required 4 h/decision aid [43]. The strategies that we implemented to speed up the review process thus cause a significant reduction in the resources required to complete the reviews compared to traditional systematic review processes that have been reported to take 32 weeks on average [6]. The approaches used to conduct reviews influence their costs, which have been reported to vary from £25,000–140,000 [approximately from C$42,000 to C$234,000] [44] and up to a quarter of a million US dollars [approximately C$336,000] [45], whereas in this study we estimated the average cost of a single review to be approximately C$11,646. Working with a team of experienced reviewers may allow limiting the rapid review costs even more. However, the provision of a training environment for inexperienced reviewers has a significant value in a university environment such as the one where we conducted this research.

The time needed to review the literature varied depending on the decision. This could be explained by variations in the number of references screened for each decision or by the number of included studies, health options, or health outcomes for each health option. DB1 and DB2 presented the highest numbers of health outcomes and were also those that required the most resources, suggesting that the number of outcomes is an important determinant of the resources required. In addition to the volume of literature, the rate of pay may also influence the costs of conducting these rapid reviews in another team. In addition, some of the costs un-related to salaries may need to be accounted for: article ordering, software, teleconferencing, information specialist, and biostatistician.

For one of the rapid reviews, i.e., on the question of the benefits and harms of preparing advanced directives and a protection mandate in case of incapacity (DB5), we found very limited evidence, which points to the need for more research in this area. It was also generally easier to identify data on benefits rather than on harms. This highlights the need for improved reporting of the negative impacts of interventions, hinting to publication bias issue [46].

### Strengths and limitations

In this study, we describe the methodology of a rapid review approach, with the aim of furthering the research on systematic reviews and on the development of shared decision-making tools. We address some of the limitations often reported with regard to rapid reviews, particularly that they lack transparency and use inappropriate reporting [7, 47, 48]. As recommended [49], we report the methodological details of our approach, to allow a careful examination of the choices we have made and reproduction of these results. This may provide a foundation for future rapid review teams, for example, when planning the resources they need [50]. Another strength of this report is the detailed documentation of each step and the careful reporting of review outcomes (time, resources).

The proposed rapid review approach. Firstly, the information specialist was involved as a consultant and did not conduct the searches, which may have affected the validity of the searches. Secondly, the experts were not blinded and so they might have refrained from making certain observations. Thirdly, the lack of quality appraisal for the qualitative studies included represents a significant limitation, especially considering the relatively important number of such studies. Fourthly, we did not create a flow diagram for each DB, to describe the number of references from each source and the reasons of exclusion.

Additional limitations concern the methods used to describe this work. First, we did not collect data to assess how the proposed approaches to accelerate review processes affected the quality of the reviews. A subsequent study could thus be conducted to compare our results to those obtained using a traditional systematic review methodology. One of the challenges of such a study would be that the current approach consists of a review of several interventions that require consideration during the course of decision-making, and this, in itself, diverges considerably from traditional systematic reviews. A second limitation is that this work relied on the reviewers’ self-reporting of the time required at each step, with risks of bias from misclassification and recall. It is uncertain to what extent such bias might impact results. The use of an online reviewing software allowing the automatic recording of the time at each review step may have been more reliable. Such reviewing software might also improve efficiency and limit the errors in data extraction, which can reach 30% in a single extraction [51]. While conversions for dichotomous outcomes were fairly straightforward, we did encounter more difficulties converting standardized mean differences to absolute risk differences. The methodology used was simple but could lead to some biases in favor of the treatment. Because of this, we believe greater care and time should be planned on these conversions, involving a biostatistician and a clinician well versed in the particular scale used.

## Conclusions

This detailed description of a rapid review approach may allow researchers in the future to choose a methodological approach and plan the time, costs, and resources needed when using this approach to produce shared decision-making tools. We found that an extensive and an experienced team is required to develop several tools within a rigid time frame. Reviewer training is critical and time-consuming, as there is a lack of resources with the appropriate expertise. An assessment of decision-making needs and rapid review of systematic reviews on the benefits and harms of multiple health options to address those needs allows for the development of decision support tools that meet users’ expectations in a timely manner. A future study is now required to test the applicability of this approach to other teams and topics.

## Additional file

Additional file 1: Search strategies used for each of the five decision boxes. (DOCX 40 kb)

## Abbreviations

AR: Absolute risks
ARD: Absolute risk difference
BPSD: Behavioral and psychological symptoms of dementia
DB: Decision box
GRADE: Grading of Recommendations Assessment, Development and Evaluation
IPDAS: International Patient Decision Aid Standards
NOS: Newcastle-Ottawa Quality Assessment Scale
PI: Principal investigator
PICO: Population, Intervention, Comparison, Outcome
RCT: Randomized controlled trial
SD: Standard deviation
SMD: Standardized mean differences
SwD: Senior living with dementia
