# Supplementary material for: Five shared decision-making tools in 5 months: use of rapid reviews to develop decision boxes for seniors living with dementia and their caregivers
Source: Syst Rev. 2017 Mar 15;6:56. doi: 10.1186/s13643-017-0446-2 (PMC5353791; doi:10.1186/s13643-017-0446-2)
Supplement: Additional file 1: — Search strategies used for each of the five decision boxes. (DOCX 40 kb) [file 13643_2017_446_MOESM1_ESM.docx]

**Additional file 1: Search strategies used for each of the five Decision Boxes**

# Decision Box 1: Choosing a support option to decrease the burden of caregivers of seniors living with dementia or to improve their quality of life

## 1. SEARCH FOR SECONDARY SOURCES OF LITERATURE (November 16^th^, 2015)

**1.1 Grey literature sources: UptoDate, Dynamed, Canadian Task Force, AHRQ, Scottish Intercollegiate Guidelines Network (SIGN), National Institute for Health and Care Excellence (NICE)**

Search strategy #1: Dementia OR Alzheimer

Search strategy #2: Caregivers OR family

**1.2 Academic databases: Cochrane Central Register of Controlled Trials (CENTRAL), PubMed (NLM), Cochrane Dementia and Cognitive Improvement review group Specialized register (ALOIS), PsycNET**

Search Strategy #3, shown here in Pubmed format:

1. Caregivers[Majr]
2. Spouses[Majr]
3. Caregiv*[Title]
4. Carer*[Title]
5. Famil*[Title]
6. Partner*[Title]
7. Spouse*[Title]
8. Dyad*[Title]
9. Friend*[Title]
10. OR/ 1-9
11. Aged[Mesh]
12. senior*[Title/Abstract]
13. elder*[Title/Abstract]
14. older[Title/Abstract]
15. geriatr*[Title/Abstract]
16. “Aged patient”[Title/Abstract]
17. “Aged person”[Title/Abstract]
18. “Aged people”[Title/Abstract]
19. “Aged women”[Title/Abstract]
20. “Aged woman”[Title/Abstract]
21. “Aged man”[Title/Abstract]
22. “Aged men”[Title/Abstract]
23. OR/11-22
24. Dementia[Mesh]
25. "Cognition disorders"[Mesh]
26. “cognitive impairment”[Title/Abstract]
27. “cognitively impaired”[Title/Abstract]
28. dement*[Title/Abstract]
29. Alzheimer*[Title/Abstract]
30. “pick’s disease”[Title/Abstract]
31. Huntington[Title/Abstract]
32. Creutzfeldt [Title/Abstract]
33. cjd[Title/Abstract]
34. binswanger[Title/Abstract]
35. lewy[Title/Abstract]
36. aphasi*[Title/Abstract]
37. OR/24-36
38. "Cost of Illness"[Mesh]
39. "Quality of life"[Mesh]
40. "Health status"[Mesh]
41. "Mental Health" [Mesh]
42. "Quality-adjusted life years"[Mesh]
43. Burden*[Title/Abstract]
44. “Illness Cost”[Title/Abstract]
45. “Neuropsychiatry symptom”[Title/Abstract]
46. “Sickness Cost”[Title/Abstract]
47. Burnout [Title/Abstract]
48. Burn-out[Title/Abstract]
49. Selfconcept[Title/Abstract]
50. Self-concept[Title/Abstract]
51. “Quality of life”[Title/Abstract]
52. Well-being[Title/Abstract]
53. wellbeing[Title/Abstract]
54. “Quality adjusted life year”[Title/Abstract]
55. QoL [Title/Abstract]
56. Distress*[Title/Abstract]
57. Wellness[Title/Abstract]
58. OR/38-57
59. AND/10, 23, 37, 58 [Filters: Humans, Reviews, English; French; Spanish]

## 2. SEARCH FOR PRIMARY SOURCES OF LITERATURE (December 15^th^, 2015 to May, 11^th^, 2016)

**2.1: Citations:** using Google Scholar, we screened the publications citing the studies included in the systematic reviews found during the search for secondary sources (step #1 above)

**2.2: Grey literature search :** using Google, we searched the websites of professional or government organizations (Ministère de la santé et des services sociaux du Québec, Alzheimer Society, l’APPUI, Institut national d’excellence en santé et services sociaux du Québec)

# Decision Box 2: Choosing a treatment to manage agitation, aggression, or psychotic symptoms in seniors living with dementia

## 1. SEARCH FOR SECONDARY SOURCES OF LITERATURE (October 1^st^, 2015)

**1.1 Grey literature sources: UptoDate, Dynamed, Canadian Task Force, AHRQ, Scottish Intercollegiate Guidelines Network (SIGN), National Institute for Health and Care Excellence (NICE)**

Search strategy #1: Dementia or Alzheimer

**1.2 Academic databases: Cochrane Central Register of Controlled Trials (CENTRAL), PubMed (NLM), Cochrane Dementia and Cognitive Improvement review group Specialized register (ALOIS), Psycnet**

Search Strategy #2, shown here in Pubmed format:

1. Aged[Majr]
2. senior*[Title/Abstract]
3. elderly[Title/Abstract]
4. older[Title/Abstract]
5. OR/1-4
6. Dementia[Majr]
7. "Cognition disorders"[Majr]
8. cognitive impairment[Majr]
9. “cognitive impairment”[Title]
10. “cognitively impaired” [Title]
11. dement*[Title]
12. Alzheimer*[Title]
13. “pick’s disease” [Title]
14. Huntington[Title]
15. Creutzfeldt[Title]
16. cjd[Title]
17. binswanger
18. lewy[Title]
19. aphasi*[Title]
20. OR/6-19
21. "Behavioral Symptoms"[Majr]
22. "neuropsychiatric symptoms"[Title]
23. ”neuro-psychiatric symptoms"[Title]
24. "psycho-behavioral symptoms"[Title]
25. "psycho-behavioural Symptoms"[Title]
26. "psychiatric symptoms"[Title]
27. "Behavioral symptoms"[Title]
28. "behavioural symptoms"[Title]
29. "psychological symptoms"[Title]
30. "disruptive behaviour"[Title]
31. "disruptive behaviour"[Title]
32. "non-cognitive symptoms"[Title]
33. "neuropsychological symptoms"[Title]
34. bpsd[Title]
35. Agitation[Title]
36. restless*[Title]
37. irrita*[Title]
38. aggression[Title]
39. “aberrant motor behav*”[Title]
40. “psychomotor activity” [Title]
41. “challenging behav*”[Title]
42. Pacing[Title]
43. sundowning[Title]
44. wander*[Title]
45. “walking about” [Title]
46. psychosis [Title]
47. hyperactivity[Title]
48. “challenging behav*”[Title]
49. OR/21-48
50. AND/5, 20, 49 [Filters: Humans, Reviews, English, French, Spanish, Published from 2000/10/01]

## 2. SEARCH FOR PRIMARY SOURCES OF LITERATURE (January 10^th^, 2016 to April, 11^th^, 2016)

**2.1: Citations:** using Google Scholar, we screened the publications citing the studies included in the systematic reviews found during the search for secondary sources (step #1 above)

**2.2: Grey literature search :** using Google, we searched the websites of professional or government organizations (Ministère de la santé et des services sociaux du Québec, Alzheimer Society, l’APPUI, Institut national d’excellence en santé et services sociaux du Québec).

**2.3 Academic Databases: Cochrane Central Register of Controlled Trials (CENTRAL), PubMed (NLM), Cinhal, PsycNET**

Search strategy #4:

1. Caregivers[Majr]
2. Spouses[Majr]
3. Caregiv*[Title]
4. Carer*[Title]
5. Famil*[Title]
6. Partner*[Title]
7. Spouse*[Title]
8. Dyad*[Title]
9. Friend*[Title]
10. OR/ 1-9
11. Aged[Mesh]
12. senior*[Title/Abstract]
13. elder*[Title/Abstract]
14. older[Title/Abstract]
15. geriatr*
16. “Aged patient”[Title/Abstract]
17. “Aged person”[Title/Abstract]
18. “Aged people”[Title/Abstract]
19. “Aged women”[Title/Abstract]
20. “Aged woman”[Title/Abstract]
21. “Aged man”[Title/Abstract]
22. “Aged men”[Title/Abstract]
23. OR/11-22
24. Dementia[Mesh]
25. "Cognition disorders"[Mesh]
26. “cognitive impairment”[Title/Abstract]
27. “cognitively impaired”[Title/Abstract]
28. dement*[Title/Abstract]
29. Alzheimer*[Title/Abstract]
30. “pick’s disease”[Title/Abstract]
31. Huntington[Title/Abstract]
32. Creutzfeldt [Title/Abstract]
33. cjd[Title/Abstract]
34. binswanger[Title/Abstract]
35. lewy[Title/Abstract]
36. aphasi*[Title/Abstract]
37. OR/24-36
38. Psychotherapy[Majr] OR Counseling[Majr] OR Psychotherap*[Title/Abstract] OR “Psychological treatment”[Title/Abstract] OR “Psychological therapy”[Title/Abstract] OR “Cognitive Therapy”[Title/Abstract] OR “Behavioral Therapy”[Title/Abstract] OR “Cognitive Behavioral Therapy”[Title/Abstract] OR “Cognitive Behavioral Intervention”[Title/Abstract] OR Counseling[Title/Abstract] OR “Relaxation therapy”[Title/Abstract] OR “Psychosocial therapy”[Title/Abstract]
39. Case management[Majr] OR Patient care planning[Majr] “Case management”[Title/Abstract]
40. Adaptation, psychological[Majr] OR Social Skills[Majr] OR Problem solving[Majr]
41. Patient Education[Majr] OR Health Education[Majr] OR Education[Majr] OR Psychoeducation[Title/Abstract] OR Problem-solving[Title/Abstract] OR Education[Title/Abstract] OR Training[Title/Abstract] OR “Psychosocial intervention” [Title/Abstract]
42. Exercise*[Majr] OR “Motor Activity” [Title/Abstract] OR Exercise*[Title/Abstract] OR “Physical Activity” [Title/Abstract] OR “Physical Training” [Title/Abstract] OR “Physical program” [Title/Abstract]
43. Respite Care[Majr] OR “Home care”[Title/Abstract] OR Home care services[Majr] “Home nursing”[Title/Abstract] OR “Home treatment”[Title/Abstract] OR “Home care service”[Title/Abstract] OR “Home environmental intervention”[Title/Abstract] OR Respite[Title/Abstract]
44. Social support[Majr] OR Self-Help groups[Majr] OR “Support group” [Title/Abstract] OR “Support program”[Title/Abstract] OR Support[Title/Abstract]
45. Telemedecine*[Title/Abstract]
46. Music*[Title/Abstract]
47. OR/38-46
48. "adverse effects" [Subheading]
49. "Drug-Related Side Effects and Adverse Reactions"[Mesh]
50. "Long Term Adverse Effects"[Mesh]
51. “adverse effect” OR “adverse effects” or “adverse event” OR “adverse events” OR “side effect” OR “side effects” OR “undesirable effect” OR “undesirable effects”
52. OR/48-51
53. AND/10,23,37,47,52

# Decision Box 3: Deciding whether or not to stop driving following a diagnosis of dementia

## 1. SEARCH FOR SECONDARY SOURCES OF LITERATURE (February 15^th^, 2016)

**1.1 Grey literature sources: UptoDate, Dynamed, Canadian Task Force, AHRQ, Scottish Intercollegiate Guidelines Network (SIGN), National Institute for Health and Care Excellence (NICE)**

Search strategy #1: Dementia or Alzheimer

**1.2 Academic databases: Cochrane Central Register of Controlled Trials (CENTRAL), PubMed (NLM), EMBASE, Cochrane Dementia and Cognitive Improvement review group Specialized register (ALOIS)**

Search Strategy #2, shown here in Pubmed format:

1. Aged[Mesh]
2. senior*[Title/Abstract]
3. elderly[Title/Abstract]
4. older[Title/Abstract]
5. Aged[Title/Abstract]
6. OR/1-5
7. Dementia[Majr]
8. cognitive impairment[Majr]
9. “cognitive impairment”[Title/Abstract]
10. “cognitively impaired”[Title/Abstract]
11. dement*[Title/Abstract]
12. Alzheimer*[Title/Abstract]
13. OR/7-12
14. Automobile driving[Majr]
15. Automobile[Title/Abstract]
16. drivin*[Title/Abstract]
17. driver*[Title/Abstract]
18. “car accident”[Title/Abstract]
19. “traffic accident”[Title/Abstract]
20. OR/14-19
21. AND/6, 13, 20 [Filters: Humans, Reviews, English, French, Spanish, Published from 1996/02/15]

## 2. SEARCH FOR PRIMARY SOURCES OF LITERATURE (March, 31^st^, 2016)

**2.1: Citations:** using Google Scholar, we screened the publications citing the studies included in the systematic reviews found during the search for secondary sources (step #1 above)

**2.2: Grey literature search :** using Google, we searched the websites of professional or government organizations (Ministère de la santé et des services sociaux du Québec, Alzheimer Society, Institut national d’excellence en santé et services sociaux du Québec, Société de l’assurance automobile du Québec)

**2.3 Academic databases: Cochrane Central Register of Controlled Trials (CENTRAL), PubMed (NLM), EMBASE, Cochrane Dementia and Cognitive Improvement review group Specialized register (ALOIS)**

Search Strategy #3, shown here in Pubmed format:

1. Aged[Mesh]
2. senior*[Title/Abstract]
3. elderly[Title/Abstract]
4. older[Title/Abstract]
5. Aged[Title/Abstract]
6. OR/1-5
7. Dementia[Mesh]
8. cognitive impairment[Mesh]
9. “cognitive impairment”[Title/Abstract]
10. “cognitively impaired”[Title/Abstract]
11. dement*[Title/Abstract]
12. Alzheimer*[Title/Abstract]
13. OR/7-12
14. Automobile driving[Mesh]
15. Automobile[Title/Abstract]
16. drivin*[Title/Abstract]
17. driver*[Title/Abstract]
18. “car accident”[Title/Abstract]
19. “traffic accident”[Title/Abstract]
20. OR/14-19
21. AND/6, 13, 20 [Filters: Clinical trials, Observational Study, Comparative Study, Humans, English, French, Spanish]

# Decision Box 4: Choosing an option to maintain or improve the quality of life of seniors living with dementia

## 1. SEARCH FOR SECONDARY SOURCES OF LITERATURE (March 1^st^, 2016)

**1.1 Grey literature sources: UptoDate, Dynamed, Canadian Task Force, AHRQ, Scottish Intercollegiate Guidelines Network (SIGN), National Institute for Health and Care Excellence (NICE)**

Search strategy #1: Dementia or Alzheimer

**1.2 Academic databases: Cochrane Central Register of Controlled Trials (CENTRAL), PubMed (NLM), Cochrane Dementia and Cognitive Improvement review group Specialized register (ALOIS)**

Search Strategy #2, shown here in Pubmed format:

1. Aged[Majr]
2. Aging[Majr]
3. senior*[Title/Abstract]
4. elderly[Title/Abstract]
5. elder[Title/Abstract]
6. older[Title/Abstract]
7. OR/1-6
8. Alzheimer disease [Majr]
9. Dementia[Mesh]
10. “cognitive function” [Title/Abstract]
11. “cognitive impairment” [Title/Abstract]
12. Dement*[Title/Abstract]
13. “advanced dementia” [Title/Abstract]
14. OR/8-13
15. Quality of life[Majr]
16. Mental Health[Majr]
17. Quality adjusted life years[Majr]
18. Self concept[Majr]
19. “Quality of life” [Title/Abstract]
20. Well-being[Title/Abstract]
21. “Quality adjusted life year” [Title/Abstract]
22. “Quality adjusted life years” [Title/Abstract]
23. QoL[Title/Abstract]
24. Distress[Title/Abstract]
25. Wellness[Title/Abstract]
26. “Satisfaction with life” [Title/Abstract]
27. “life satisfaction” [Title/Abstract]
28. “sense of self-worth” [Title/Abstract]
29. OR/15-28
30. AND/7, 14, 29 [Filters: Humans, Reviews, English, French, Spanish, Published from 2011/03/01]

## 2. SEARCH FOR PRIMARY SOURCES OF LITERATURE (March 31^st^, 2016 to July, 20^th^, 2016)

1. Aged[Mesh]
2. Aging[Mesh]
3. senior*[Title/Abstract]
4. elderly[Title/Abstract]
5. elder[Title/Abstract]
6. older[Title/Abstract]
7. OR/1-6
8. Alzheimer disease [Mesh]
9. Dementia[Mesh]
10. “cognitive function” [Title/Abstract]
11. “cognitive impairment” [Title/Abstract]
12. Dement*[Title/Abstract]
13. “advanced dementia” [Title/Abstract]
14. OR/8-13
15. Quality of life[Majr]
16. Mental Health[Majr]
17. Quality adjusted life years[Majr]
18. Self concept[Majr]
19. “Quality of life” [Title]
20. Well-being[Title]
21. “Quality adjusted life year” [Title]
22. “Quality adjusted life years” [Title]
23. QoL[Title]
24. Distress[Title]
25. Wellness[Title]
26. “Satisfaction with life” [Title]
27. “life satisfaction” [Title]
28. “sense of self-worth” [Title]
29. OR/15-28
30. AND/7, 14, 29 [Filters: Clinical trials, Humans,]

**2.2: Grey literature search**

2.2.1: Using the Google search engine: Websites of professional or government organizations (Ministère de la santé et des services sociaux du Québec, Alzheimer Society, Institut national d’excellence en santé et services sociaux du Québec)

2.2.2: Using google Scholar: find publications citing the studies included in the included reviews.

# Decision Box 5: Deciding whether to prepare advanced directives and a protection mandate following a diagnosis of dementia.

## 1. SEARCH FOR SECONDARY SOURCES OF LITERATURE (March 1^st^, 2016)

**1.1 Grey literature sources: UptoDate, Dynamed, Canadian Task Force, AHRQ, Scottish Intercollegiate Guidelines Network (SIGN), National Institute for Health and Care Excellence (NICE)**

Search strategy #1: Dementia or Alzheimer

**1.2 Academic databases: Cochrane Central Register of Controlled Trials (CENTRAL), PubMed (NLM), EMBASE, Cochrane Dementia and Cognitive Improvement review group Specialized register (ALOIS)**

Search Strategy #2 shown here in Pubmed format:

1. Aged[Mesh]
2. senior*[Title/Abstract]
3. elderly[Title/Abstract]
4. older[Title/Abstract]
5. OR/1-6
6. Alzheimer disease [Majr]
7. Dementia[Majr]
8. dement*[Title/Abstract]
9. alzheimer[Title/Abstract]
10. “vascular dementia”[Title/Abstract]
11. “pick’s disease”[Title/Abstract]
12. “huntington creutzfeldt”[Title/Abstract]
13. cjd[Title/Abstract]
14. binswanger[Title/Abstract]
15. lewy[Title/Abstract]
16. “cognitive impairment”[Title/Abstract]
17. “cognitive impairments”[Title/Abstract]
18. OR/6-17
19. “legal capacity” [Title/Abstract]
20. aptness[Title/Abstract]
21. “protection policy” [Title/Abstract]
22. “legal assessment” [Title/Abstract]
23. guardianship[Title/Abstract]
24. fiduciary[Title/Abstract]
25. “surrogate management”[Title/Abstract]
26. conservatorship[Title/Abstract]
27. “living will”[Title/Abstract]
28. “advance care planning”[Title/Abstract]
29. “power of attorney”[Title/Abstract]
30. OR/19-29
31. AND/5, 18, 30 [Filters: Humans, English, French, Spanish, Published from 2006/03/01]

## 2. SEARCH FOR PRIMARY SOURCES OF LITERATURE (March 1^st^, 2016 to April 4^th^, 2016)

**2.1. Citations:** using Google Scholar, we screened the publications citing the studies included in the systematic reviews found during the search for secondary sources (step #1 above)

**2.2. Grey literature search :** using Google, we searched the websites of professional or government organizations (Ministère de la santé et des services sociaux du Québec, Alzheimer Society, Institut national d’excellence en santé et services sociaux du Québec, Curateur public du Québec)
